# ‘Hacıhaliloğlu’ apricot under simulated drought: morphological, physiological, biochemical, and flower biology responses

**DOI:** 10.1186/s12870-025-07983-9

**Published:** 2026-01-03

**Authors:** Muzaffer İpek, Şeyma Arıkan, Duran Yavuz, Ahmet Eşitken, Lütfi Pırlak, Hüseyin Karlıdağ

**Affiliations:** 1 Department of Horticulture, Faculty of Agriculture, Selçuk University, Konya, Türkiye; 2 Department of Agricultural Structures and Irrigation, Faculty of Agriculture, Selçuk University, Konya, Türkiye; 3 Department of Horticulture, Faculty of Agriculture, Malatya Turgut Özal University, Malatya, Türkiye

**Keywords:** Apricot, Drought stress, Antioxidant enzymes, Flower bud development, Physiological traits

## Abstract

Apricot is one of Türkiye’s most important horticultural crops, accounting for approximately 21% of global production. The Hacıhaliloğlu cultivar, responsible for 90% of the country’s dried apricot exports, is cultivated in Malatya, where declining precipitation due to climate change poses a significant threat to sustainable production. This study, conducted from 2021 to 2023, aimed to evaluate the effects of different drought irrigation regimes on the morpho-physiological and biochemical characteristics of Hacıhaliloğlu apricot trees under water stress. Four-year-old T-budded saplings were grown in pots and subjected to monthly irrigation treatments during the post-harvest period.

The results revealed that drought stress significantly inhibited shoot elongation, reduced leaf size and dry matter accumulation, and impaired pistil development. The T_0_ treatment (full irrigation) consistently outperformed all other regimes in terms of shoot length, pistil length, specific leaf weight, and relative leaf dry weight. Drought-exposed trees, especially those under rainfall-only or late irrigation conditions (T_1_, T_7_, T_8_), exhibited increased oxidative damage, as indicated by elevated levels of membrane permeability, H₂O₂, MDA, and antioxidant enzyme activities (CAT, POD, SOD). Moreover, leaf water potential and chlorophyll content declined under prolonged stress conditions.

These findings emphasize that irrigation during critical developmental stages particularly July and August, when flower bud differentiation and vegetative growth overlaps is vital for preserving productivity and physiological integrity in apricot trees. Strategic water management in arid and semi-arid regions can mitigate the negative effects of drought stress and enhance tree performance even under limited water availability.

## Introduction

**Fig. 1:**
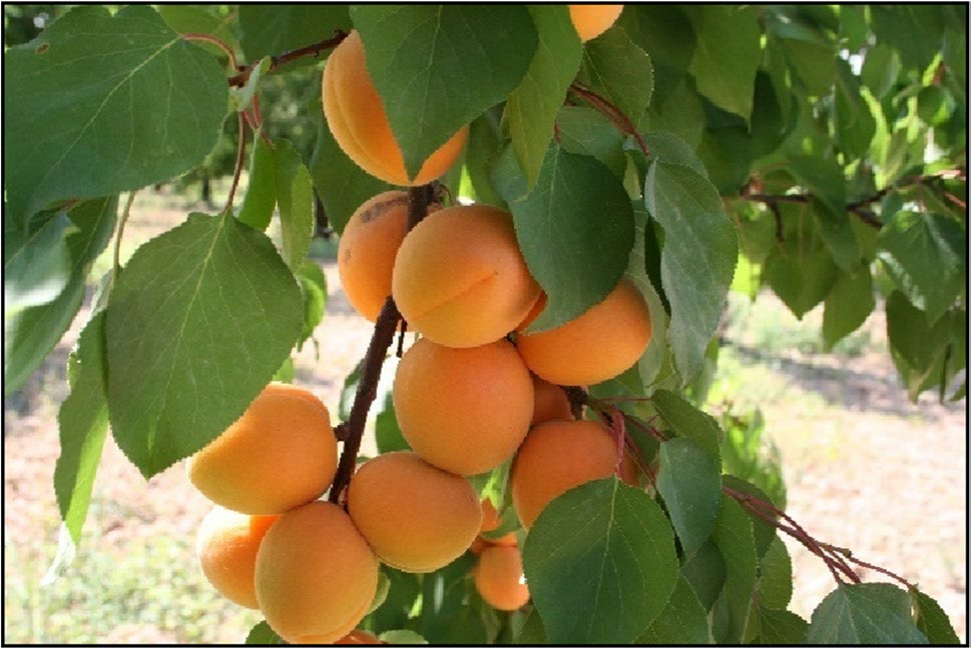
The Hacıhaliloğlu cultivar's fruits (from Prof. Dr. Hüseyin Karlıdağ)

**Fig. 2:**
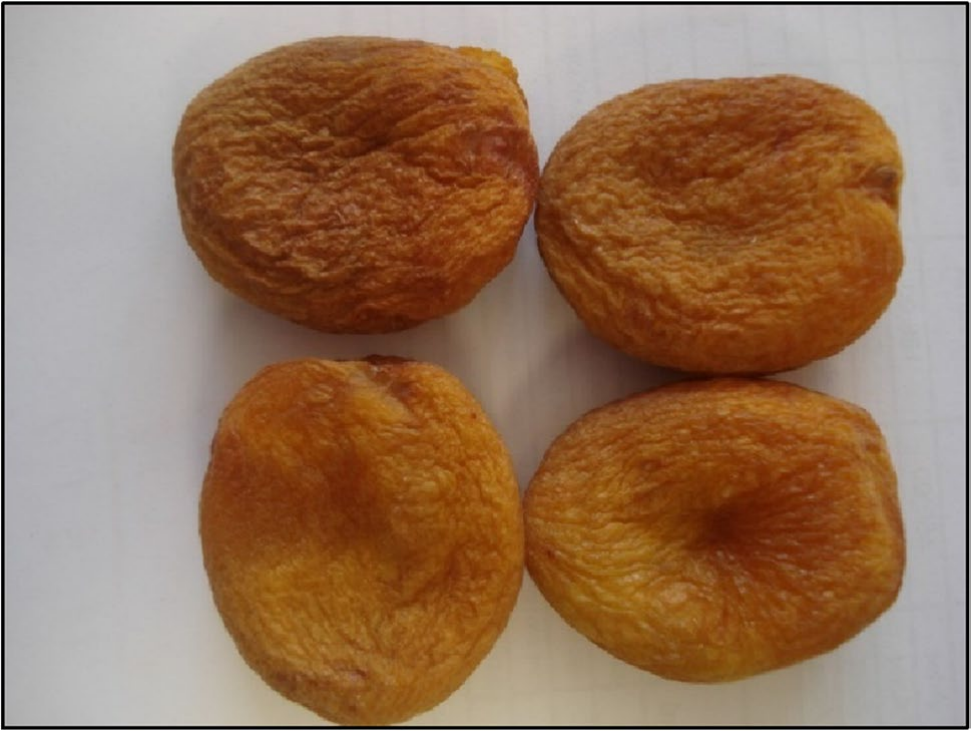
Sun dried fruit of the Hacıhaliloğlu cultivar (from Prof. Dr. Hüseyin Karlıdağ)

Apricot (*Prunus armeniaca* L.) originates from a broad region extending into the mountainous areas of northern and northeastern China, while Anatolia is recognized as a secondary center of diversity due to its long history of cultivation [1]. Türkiye is a major global apricot producer, yielding about 803,000 tons annually and accounting for roughly 21% of worldwide production [2, 3]. Within Türkiye, Malatya contributes nearly 41% of national apricot output, with orchards dominated by the Hacıhaliloğlu (Figs. 1 and 2) and Kabaaşı cultivars, which are preferred for drying due to their high dry matter content (20–25%) and low acidity (0.16–0.23%) [4].

Although the region’s climate generally supports the cultivation of high-quality apricot cultivars, growers in Malatya frequently face irrigation shortages during summer because of the semi-arid climate and irregular rainfall distribution. In some years, only 2–3 irrigations are feasible, which is insufficient to meet tree water requirements during vegetative growth and flower bud differentiation. This limitation has become more pronounced in recent years, as water scarcity particularly due to reduced autumn rainfall [5] has intensified drought stress across the production area.

Under inadequate irrigation and nutrient availability, apricot trees exhibit various floral abnormalities, including incomplete pistils and reduced bud development, leading to substantial declines in yield and fruit quality [6, 7]. Although apricot trees are generally considered to have relatively low water requirements, their drought tolerance is limited, and they remain vulnerable during key phenological stages such as floral bud formation and differentiation [8, 9]. This developmental sensitivity aligns with observations in temperate fruit species, where flower bud initiation typically begins 30–45 days after flowering and continues throughout the growing season [10]. Adequate irrigation during this window is therefore essential, as severe post-harvest water stress reduces floral bud quality and quantity, pollen viability, and ultimately fruit set [11–15].

Long-term meteorological records further emphasize the vulnerability of apricot cultivation in the region. Annual and seasonal precipitation in Malatya has shown a declining trend, with 16 of the past 28 years falling below the historical average of 374.2 mm [16], thereby exacerbating drought stress during critical phenological stages.

Against this background, the present study evaluates the morphological, physiological, and biochemical responses of the Hacıhaliloğlu apricot cultivar to year-round drought, with particular emphasis on identifying the phenological stages especially post-harvest vegetative growth and floral bud differentiation that are most sensitive to water scarcity.

## Materials and methods

### Plant materials

In the research, 4-year-old Hacıhaliloğlu apricot variety grafted with “T bud” on seedling was used as plant material. The young apricot trees were cultivated by a commercial sapling producer in Malatya province, nurtured until they reached the age of 4, and subsequently relocated to Konya province (38.030255 N, 32.509354E), where the research was conducted. The Hacıhaliloğlu apricot trees are cultivated in 150-Liter plastic pots containing a mixture of orchard soil, river sand, and manure at a ratio of 4:2:1 (v: v:v).

### Experimental design

The study was conducted between 2021 and 2023 using a total of 90 young apricot trees. The experimental design included ten different irrigation treatments, arranged in three replications, with each replication consisting of three young apricot trees.

To control environmental conditions and ensure the fulfillment of chilling requirements, the young apricot trees were grown in a greenhouse from November to June. Between July and October, the trees were transferred outside the greenhouse to the project area under 20% shading.

The irrigation strategy of the experiment was developed based on the analysis of long-term precipitation data from the Malatya region (> 100 years). This analysis identified the period from March 2016 to February 2017 as the driest year within the dataset, and therefore this period was selected as the reference rainfall regime for the study. The primary aim of the experiment was to determine the most appropriate irrigation timing for apricot trees subjected to drought stress during the post-harvest period. For this purpose, a monthly irrigation schedule was established according to the precipitation data of the 2016–2017 period.

In this plan, the T_0_ treatment represented the control group, which received full irrigation throughout the entire year, with soil moisture in the root zone restored to field capacity whenever necessary. The T_1_ treatment was irrigated solely according to the monthly precipitation amounts recorded for Malatya between March 2016 and February 2017 (Table 1) and thus represented the drought-stress (rain-fed) regime.

**Table 1:** Malatya received monthly rainfall from March 2016 to February 2017 [16]

March	April	May	June	July	August	September	October	November	December	January	February	Total
12.5	6.1	43.3	7.6	14.3	5.3	11.7	1.1	13.3	59.3	21.1	0.2	**195.8**

Treatments T_2_ to T_8_ received the same Malatya monthly rainfall in all months as T_1_, except in their designated month, when the pots were additionally irrigated until the soil water content in the root zone reached field capacity. Detailed information on the irrigation schedule is presented in Table 2.

**Table 2:** Irrigation treatments applied to ‘Hacıhaliloğlu’ apricot trees under Malatya rainfall conditions

Treatments	Irrigation regime	Explanation
T0 (Full irrigation)	Full irrigation based on soil moisture (field capacity)	Soil moisture in the root zone was monitored throughout the season and irrigation was applied whenever necessary to restore soil water content to field capacity.
T_1_ (Rain-fed)	Rain-fed, based on Malatya monthly rainfall	No supplemental irrigation was applied; trees received only the monthly rainfall recorded for Malatya (Table 1).
T_2_	Malatya rainfall (all months) + irrigation to field capacity in July	In all months, trees received only the Malatya monthly rainfall, except in July, when the pots were additionally irrigated until the soil in the root zone reached field capacity.
T_3_	Malatya rainfall (all months) + irrigation to field capacity in August	In all months, trees received only the Malatya monthly rainfall, except in August, when the pots were additionally irrigated until the soil in the root zone reached field capacity.
T_4_	Malatya rainfall (all months) + irrigation to field capacity in September	In all months, trees received only the Malatya monthly rainfall, except in September, when the pots were additionally irrigated until the soil in the root zone reached field capacity.
T_5_	Malatya rainfall (all months) + irrigation to field capacity in October	In all months, trees received only the Malatya monthly rainfall, except in October, when the pots were additionally irrigated until the soil in the root zone reached field capacity.
T_6_	Malatya rainfall (all months) + irrigation to field capacity in November	In all months, trees received only the Malatya monthly rainfall, except in November, when the pots were additionally irrigated until the soil in the root zone reached field capacity.
T_7_	Malatya rainfall (all months) + irrigation to field capacity in December	In all months, trees received only the Malatya monthly rainfall, except in December, when the pots were additionally irrigated until the soil in the root zone reached field capacity.
T_8_	Malatya rainfall (all months) + irrigation to field capacity in January	In all months, trees received only the Malatya monthly rainfall, except in January, when the pots were additionally irrigated until the soil in the root zone reached field capacity.

In all treatments, the seasonal irrigation amount was first calculated on a monthly basis and then split into weekly applications to provide uniform water availability and to avoid abrupt changes in soil moisture. Prior to the experiment, the water-holding capacity of the root-zone soil (0–60 cm) was determined using a TEROS-12 sensor (Meter Group Inc., NE, USA), and these values were used to schedule irrigation events consistently across treatments.

**Fig. 3:**
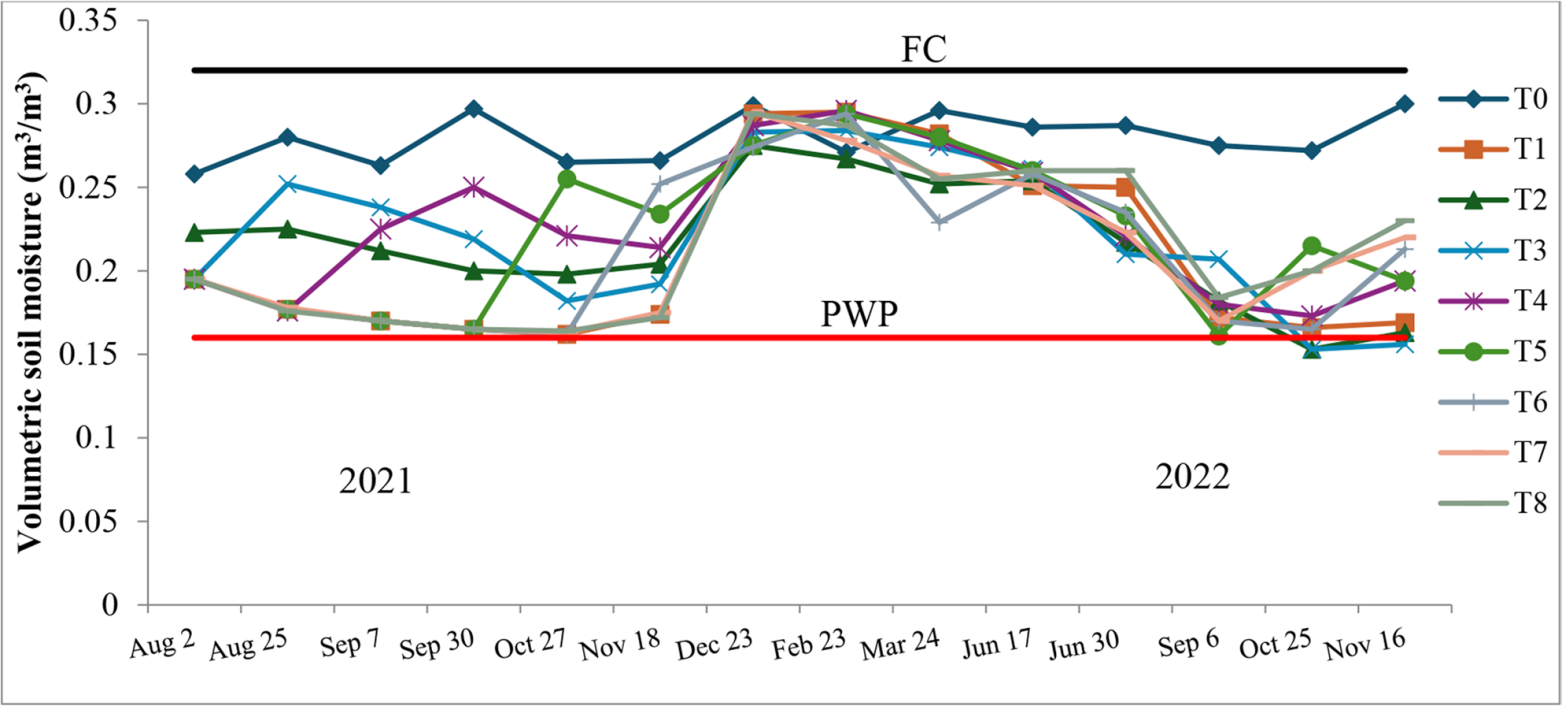
Seasonal course of volumetric soil moisture (m³ m⁻³) in the root zone (0–60 cm) of apricot trees under different post-harvest irrigation treatments (T0–T8) during the 2021–2022 experimental period. The horizontal lines indicate field capacity (FC ≈ 0.30 m³ m⁻³) and the permanent wilting point (PWP ≈ 0.16 m³ m⁻³)

Volumetric soil moisture dynamics during the 2021 and 2022 seasons are presented in Fig. 3. Throughout the trial, soil moisture in the root zone fluctuated between the permanent wilting point (PWP) and field capacity (FC). In the water-stressed treatments (T1–T8), soil water content frequently declined from near FC to values close to PWP during the post-harvest period, particularly from August to November, and remained near PWP for extended periods. On this basis, these treatments were classified as experiencing severe water stress, whereas soil moisture in the fully irrigated control (T_0_) stayed close to FC for most of the season and approached FC in all treatments during the winter months.

### Morphological analysis

The study examined several morphological traits, including as annual shoot length (cm), annual shoot diameter (mm), rootstock diameter (mm), scion diameter (mm), leaf area (cm^2^), specific leaf weight (mg cm^−2^), and leaf dry weight (%). Measurements of annual shoot length, annual shoot diameter, rootstock diameter, and scion diameter were taken using digital calipers (Mitutoyo 500–507-10) during the dormant season, after defoliation. For the measurement of the leaf’s fresh and dry weight, scales with a precision of 0.001 were utilized. The leaf area was quantified using the Photoshop CS6 extended software. The leaf areas, captured in photographs using a ruler, were analyzed using Adobe Photoshop CS6 Extended software. The pixel values of the leaf areas were subsequently converted to square centimeters. In June and July, mature leaves located in the middle of each apricot tree shoot were collected and subsequently measured for their surface area using Photoshop CS6 Extended. The specific leaf weight calculated using the algorithm provided by [17].

$$\:\mathrm{SLW\:(mg}\:{\mathrm{cm}}^{\mathrm{-2}}\mathrm{)=}\frac{\mathrm{leaf\:}\mathrm{dry\:}\mathrm{w}\mathrm{e}\mathrm{ight}}{\mathrm{leaf\:area}}$$

In order to calculate the leaf dry weight (LDW) percentage, mature leaf samples were collected from apricot trees. The fresh weight of the samples was measured, and then they were subjected to drying in an oven at a temperature of 72 °C for a duration of 48 h. The final weight of the dried samples was recorded [18]., presented an equation for determining the weight of dried leaves.

$$\mathrm{LDW}\left(\%\right)=\frac{\mathrm{dry}\;\mathrm{leaf}\;\mathrm{weight}}{\mathrm{fresh}\;\mathrm{leaf}\;\mathrm{weight}}\times100$$

### Pistil development

The growth of the pistils (at the anthesis stage) on graph paper was examined and captured using a Leica S9i stereo microscope, manufactured by Leica Microsystems in Singapore. This digital microscope has a zoom range of 0.61x-5.5x and a total magnification of 6.1x-55x when used with 10x eyepieces. The 10 MP camera is equipped with a 1080p HD video display.

### Physiological analysis

The study examined several physiological indicators, including relative leaf water content (RLWC), leaf water potential (LWP), membrane permeability (MP), and SPAD value. These measurements were planned in accordance with the irrigation schedule, where the total monthly irrigation amount was divided into weekly applications. Physiological measurements were conducted four times within each monthly irrigation cycle, with one measurement taken before each weekly irrigation event. In this approach, data were collected prior to every weekly water application in order to assess the physiological status of the trees at consistent intervals throughout the month. The four measurements obtained during each month were averaged to calculate the monthly value for each physiological parameter. This strategy ensured that the physiological responses were accurately represented by integrating the effects of the weekly distributed irrigation across the entire monthly period.

To ascertain the RLWC, fully matured leaves obtained from both control and stress-treated plants were initially assessed for their fresh weight. Subsequently, the samples were immersed in a culture tube containing double-distilled water for a duration of 4 h at ambient temperature to determine their turgid weight (hydration). The weight of the swollen leaves was measured, and then the leaves were dehydrated in an oven at a temperature of 65 °C for a duration of 48 h. The RLWC % was calculated using the following formula:

$$\mathrm{RLWC}\left(\%\right)=\frac{\mathrm{FW}-\mathrm{DW}}{\mathrm{TW}-\mathrm{DW}}\times100$$

FW refers to the weight of a leaf when it is fresh, DW refers to the weight of a leaf when it is dry, and TW refers to the weight of a leaf when it is turgid [19].

In order to measure leaf water potential (LWP), a portable pressure chamber device (SKPM 1405, Skye Instruments, United Kingdom) was used. The measurements were taken one day before and seven days after each irrigation, specifically between 11:00 and 13:00. Five fully developed leaves from the middle section of the annual shoot were selected for the measurements, following the method described by [20].

The MP was examined using the methodology outlined by [21]. Electrolyte leakage was utilized to determine MP. Leaf samples were obtained and divided into three segments, each having a diameter of one cm. The leaf disks were placed into tubes containing 10 mL of distilled water. The samples were stored at a temperature of 25 °C on a shaker (IKA, KS 3000ic, China) set at a speed of 150 rpm for a duration of 24 h. The solution was assessed for electrical conductivity (EC1 value) using a VWR CO 3000 H meter from Germany after being shaken. The identical specimens were subjected to a Hirayama HVA-85 autoclave (Hirayama AT-HVA-85, Saitama Japan) at a temperature of 121 °C for a duration of 20 min. The temperature of the solution (25 ± 1 °C) was reduced, and then its electrical conductivity was measured using an EC meter, specifically for EC2. The MP was computed with the following formula and shown as a percentage.

$$\mathrm{MP}\left(\%\right)=\frac{{\mathrm{EC}}_1}{{\mathrm{EC}}_2}\times100$$

The leaf’s chlorophyll content was quantified using the SPAD-502 device manufactured by Konica Minolta in Osaka, Japan. The SPAD-502 quantifies the proportionate level of chlorophyll by assessing the absorbance of the leaf in two specific wavelength ranges, namely the red and near-infrared regions. The meter utilizes the two absorbances to compute a numerical SPAD value that is directly related to the quantity of chlorophyll in the leaf.

### Biochemical analysis

#### H_2_O_2_ content

Phosphate buffer with a pH of 7.0 and potassium iodide (KI) solutions at a concentration of 1 mM were added to the supernatant, which had a volume of 0.5 mL. The permeability of the mixture was quantified at a wavelength of 390 nm, and the concentration of H_2_O_2_ (mol g^−1^ Fresh Tissue) was estimated using a standard graph with the equation y = 0.0353x-0.0807 and a coefficient of determination (y = 0.0353x-0.0807, R^2^ = 0.9706).

#### MDA content

The quantity of lipid peroxidation was determined by measuring the amount of malondialdehyde equivalents [22]. Following the process of homogenizing the fresh leaves, which weighed 0.2 g, in a solution of 3 mL of 0.1% TCA (trichloroacetic acid), the resulting mixture was subjected to centrifugation at a speed of 10.000 rpm for a duration of 10 min. The supernatant was added to a solution containing 4 mL of 20% TCA and 1 mL of 0.5% thiobarbituric acid (TBA). The samples underwent centrifugation at a speed of 10,000 rpm for a duration of 5 min, after a heating process at 95 °C for 30 min, and subsequent cooling in an ice bath. The spectrophotometric absorbance was measured at a wavelength of 532 nm for MDA, whereas the absorbance at 600 nm was utilized to ascertain non-specific absorption for MDA-TBA combination.

$$\begin{aligned} & \mathrm{MDA}\;\mathrm{content}\;\left(\mathrm{mmol}\;\mathrm{kg}^{-1}\right)\\&=\frac{\left({\mathrm{ABS}}_{532}-{\mathrm{ABS}}_{600}\right)\times\mathrm V}{\left(\mathrm\varepsilon\times\mathrm L\times\mathrm W\right)} \end{aligned}$$

The optical density values at 532 and 600 nm are represented by ‘ABS532’ and ‘ABS600’, respectively. ‘V’ represents the volume of the total extraction mixture in liters, ‘L’ represents the length of the absorbance path in centimeters, ‘ε’ represents the extinction coefficient (155 mmol kg^−1^), and ‘W’ represents the fruit weight used in kilograms.

#### Antioxidant enzymes activity

To standardize the fresh leaf samples (1 g), we used 5 mL portions of 0.1 M potassium phosphate (KH_2_PO_4_, pH: 7.0) solution containing 1 mM EDTA and 1% polyvinylpyrrolidone (PVP). Following the transfer of the homogenate into centrifuge tubes, it underwent centrifugation at a speed of 15.000 rpm and a temperature of 4 °C for a duration of 15 min. The supernatant was used as a source to determine the activity levels of the CAT, SOD, and POD antioxidant enzymes [23].

The measurement of CAT activity was conducted using [24] protocol. The measurement of SOD activity involves the photochemical reduction of nitroblue tetrazolium chloride (NBT), as described by [25] and [26]. The activity of peroxidase (POD) can be determined by monitoring the increase in absorbance at 470 nm caused by the interaction between guaiacol and H_2_O_2_, as described by [23].

### Statistical analysis

The experiment was conducted between 2021 and 2023, and the data collected over this period were pooled across three years to provide a comprehensive evaluation of treatment effects. Statistical analyses were performed using analysis of variance (ANOVA), and mean comparisons were conducted with the Tukey multiple comparison test at a significance level of *P* < 0.05. All statistical procedures were conducted using OriginPro 2022 software (OriginLab-Origin and OriginPro-Data Analysis and Graphing Software; OriginLab Corporation, Northampton, MA, USA).

## Results

The results of this study demonstrate that limited irrigation, based on rainfall levels observed during drought periods, impairs vegetative growth, causes irregularities in pistil development, and negatively affects the physiological and biochemical characteristics of young apricot trees. These findings highlight the critical importance of adequate water supply for sustaining growth and reproductive development under drought stress conditions.

### Determination of the morphological properties

**Fig. 4:**
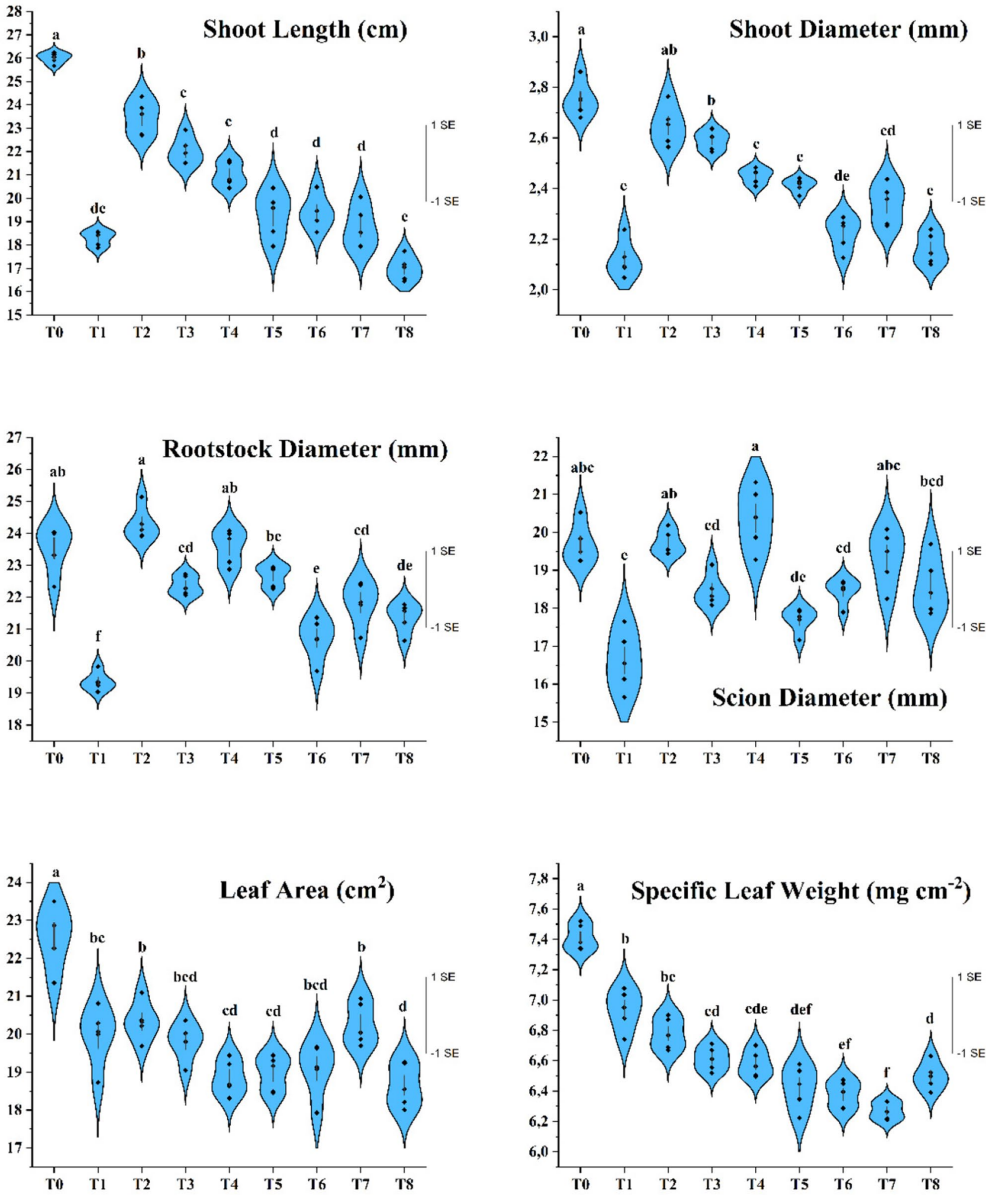
Irrigation treatments affected morphological properties of young apricot trees (cv. Hacıhaliloğlu). Values are presented as mean ± SE (n = 5), with samples taken from the pooled canopy of all trees within each treatment (9 trees in total), and data pooled across 2021–2023. Different letters indicate statistically significant differences among irrigation treatments according to Tukey’s multiple comparison test (P ≤ 0.05)

Irrigation treatments significantly affected shoot length, with the longest annual shoots in T_0_ (26.00 cm) and the shortest in T_8_ (16.99 cm) and T_1_ (18.28 cm) (Fig. 4). Irrigation to field capacity consistently promoted greater shoot elongation compared to rainfall-based regimes. The poorest shoot growth occurred under the drought-dependent T_1_ treatment and in T_8_, where irrigation applied in January did not support recovery. In contrast, additional irrigation in July (T_2_) produced shoot lengths closest to the fully irrigated control.

The reduction in shoot growth under water-limited conditions reflects restricted cell division and expansion caused by decreased tissue water content [27], hormonal imbalance particularly reduced cytokinin transport and impaired nutrient uptake [28]. Drought also reduces photosynthetic capacity, largely due to the sensitivity of PSII to water deficit [29], leading to carbohydrate limitation and suppressed shoot elongation. These results align with previous reports showing drought-induced reductions in vegetative growth in Pistacia species [30–32], almond [33, 34], fig [35], peach [36], plum [37], olive [38, 39] and with studies linking reduced photosynthesis and carbon supply to inhibited growth [12, 40, 41].

For annual shoot diameter (SD), the greatest values occurred in T_0_ (2.75 mm) and T_2_ (2.64 mm), while the lowest were recorded in T_6_ (2.22 mm), T_8_ (2.16 mm) and T_1_ (2.11 mm) (Fig. 4). January irrigation (T_8_) did not improve SD, whereas treatments reaching field capacity in other months enhanced radial growth. Rainfall-based regimes produced the smallest rootstock and scion diameters, whereas irrigation in July, September and December achieved higher values, underscoring the importance of both timing and water availability.

Stem diameter is a sensitive indicator of plant water status [42], and limited water uptake coupled with high transpiration demand commonly suppresses radial growth [43]. Similar reductions in diameter under drought have been documented in apple, fig, pomegranate, olive and Pistacia khinjuk [32, 43–45], supporting the close relationship between irrigation strategy and structural development in young apricot trees.

Leaf area was strongly influenced by irrigation treatments, with the largest values in T_0_ (22.56 cm²), followed by T_2_ (20.34 cm²) and T_7_ (20.27 cm²), and the smallest in T_8_ (18.65 cm²) (Fig. 3). Drought-related treatments clearly reduced leaf area, consistent with findings in pistachio [46–48] and almond [17, 49, 50]. Smaller leaves under drought reflect reduced turgor, fewer expanding leaves and lower rates of mitosis and cell enlargement [51, 52, 53]. Functionally, reduced leaf size limits transpiration and helps protect the photosynthetic apparatus from excess light and photoinhibition [54, 55]. Accordingly, the decline in leaf area observed here represents both growth limitation and an adaptive morphological response to water deficit.

Specific leaf weight (SLW) also varied among treatments, with the highest value in T_0_ (7.41 mg cm⁻²) and lower values in T_5_ (6.42 mg cm⁻²), T_6_ (6.37 mg cm⁻²) and T_7_ (6.27 mg cm⁻²) (Fig. 3). Higher SLW under well-watered conditions indicates thicker leaves with greater structural and photosynthetic investment, whereas drought-related reductions suggest thinner leaves with lower dry matter per unit area. Previous studies report both decreases and increases in SLW depending on species and stress intensity declines in Pistacia vera [56] and avocado [57], and mixed responses in olive [42], almond [17], peach [58], fig [35] and others [59]. The overall reduction in SLW in our study indicates that young Hacıhaliloğlu trees primarily respond to water deficit by producing thinner leaves with reduced dry matter accumulation.

### Determination of the pistil length

**Fig. 5:**
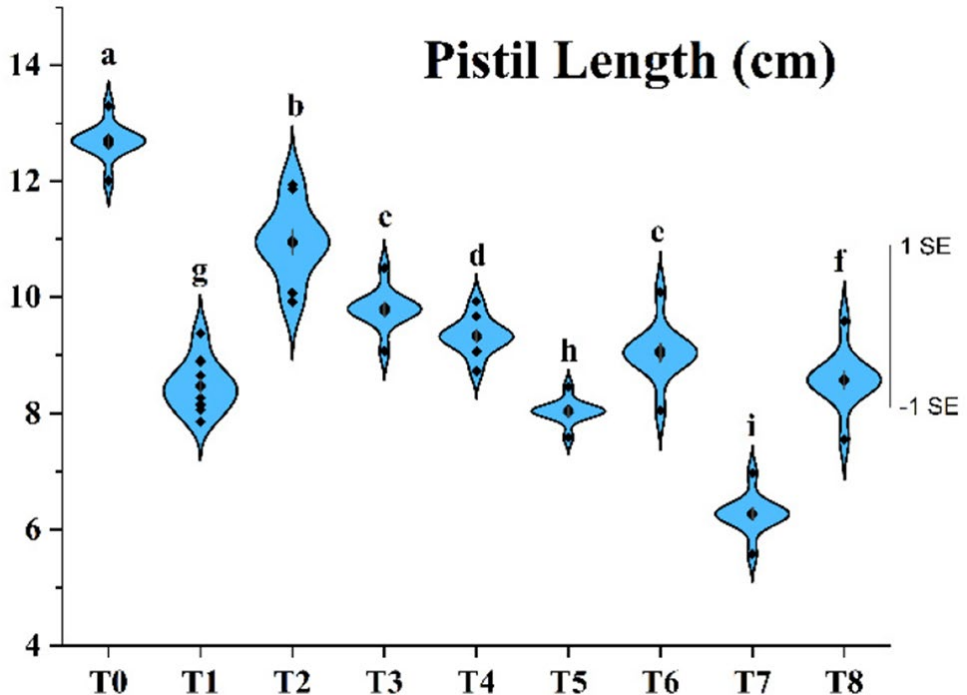
Effects of the irrigation treatments on pistil length. Values are presented as mean ± SE (n = 10), with samples taken from the pooled flower set of all trees within each treatment (9 trees in total), and data pooled across 2021–2023. Different letters indicate statistically significant differences among irrigation treatments according to Tukey’s multiple comparison test (P ≤ 0.05)

The pistil length of apricot flowers varied markedly depending on irrigation timing (Fig. 5). The longest pistils were obtained under the fully irrigated T_0_ treatment (12.68 mm), whereas the shortest (6.27 mm) occurred in T_7_, where irrigation was applied only in December. This strong reduction reflects the developmental physiology of apricot flowers: floral bud initiation, differentiation and organogenesis occur during summer and early autumn and are highly sensitive to water availability during this period [60]. Once these developmental stages are completed, later irrigation cannot compensate for earlier disruptions in pistil cell division and expansion. Because pistil differentiation and elongation occur well before winter, irrigation applied only in December is too late to influence pistil development. These findings are consistent with reports showing that water availability during organ differentiation is decisive for floral organ size and integrity [60, 61].

Irrigation applied in July and August coinciding with the post-harvest period substantially increased pistil length relative to rainfall-based or delayed irrigation regimes. These months represent a critical window for floral bud differentiation in apricot, during which water deficits strongly suppress meristematic activity and reduce cell division and expansion [12, 14, 62]. Under such conditions, reduced photosynthetic carbon gain and altered source–sink relationships limit carbohydrate allocation to developing floral meristems, constraining pistil cell expansion and limiting final pistil size. The present findings support this mechanism, as treatments irrigated in July and/or August produced pistils more similar to those of T_0_ trees.

Although pistil lengths in T_2_ and T_8_ did not fully reach T_0_ values, they were clearly greater than in T_1_, indicating that even limited irrigation during the post-harvest phase can partially mitigate drought stress and support reproductive organ development. This suggests that floral tissues maintain some degree of developmental plasticity during the early stages of organ initiation.

Supplementary Fig. 7 provides visual comparisons of pistil morphology across treatments. These images confirm the anatomical effects of drought during critical developmental periods: stigmas appear underdeveloped and styles visibly shorter in T_7_ and T_5_ compared with T_0_ or T_2_. Such morphological constraints may impair pollination and fertilization efficiency, ultimately reducing fruit set and yield potential.

Despite some treatments receiving similar total water amounts, substantial differences in pistil length were observed, demonstrating that water availability during specific developmental windows is more decisive than annual irrigation totals. These contrasting responses align with previous observations that summer water stress alters floral differentiation and bud development in apricot (Bartolini et al., 2020). Overall, our results indicate that both the amount and timing of irrigation are critical for pistil development, and that supplemental irrigation during July and August plays a key role in enhancing reproductive success under drought-prone conditions.

### Determination of the physiological properties

**Fig. 6:**
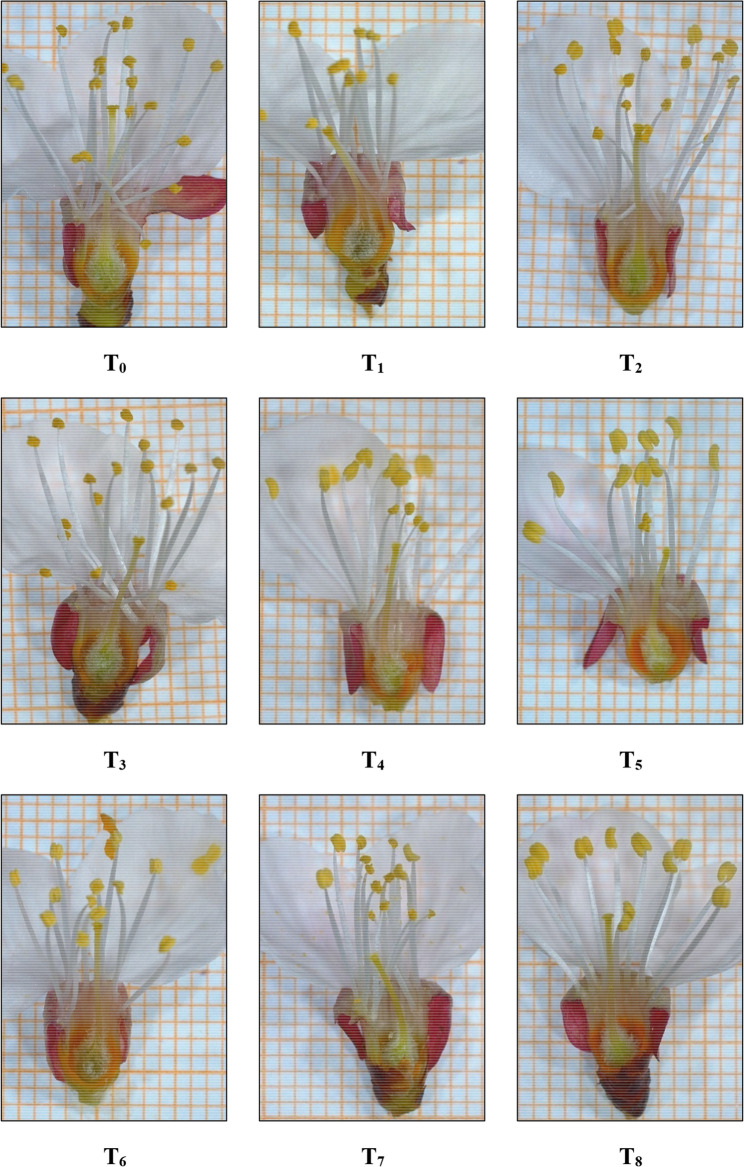
Impact of irrigation treatments on the length of pistils (graph paper used in the sub-background is one mm^2^ each square) in young apricot trees

Leaf water potential exhibited distinct differences among irrigation treatments (Fig. 6). T_0_ showed the highest water status (–3.06 MPa), whereas T_8_ had the lowest (–4.38 MPa). Since drought treatments began in early July, prolonged and intensified water deficit resulted in a progressive decline in leaf water potential, while supplemental irrigation provided partial recovery. These results confirm that both the timing and continuity of water supply strongly shape the hydraulic status of young apricot trees. Similar drought-induced reductions in leaf water potential have been reported in multiple fruit species [63–65]. Persistent decreases can disrupt internal water balance, reduce turgor and impair key cellular processes essential for vegetative and reproductive development, including pistil formation.

Cell membrane permeability (MP) also varied significantly, with the lowest value in T_0_ (8.87%) and the highest in T_8_ (19.06%) (Fig. 6). As a well-established indicator of cellular damage [66], increased MP under drought reflects oxidative stress–induced ROS accumulation, which attacks membrane lipids and proteins, causing structural deterioration [67, 68]. In this study, prolonged drought elevated MP, indicating weakened membrane stability and greater solute leakage, while supplemental irrigation helped maintain lower MP values. Similar drought-related increases in membrane permeability have been documented in pear, apple, mulberry and olive [38, 69–71, 72], supporting the protective role of adequate irrigation in limiting oxidative membrane damage.

**Fig. 7:**
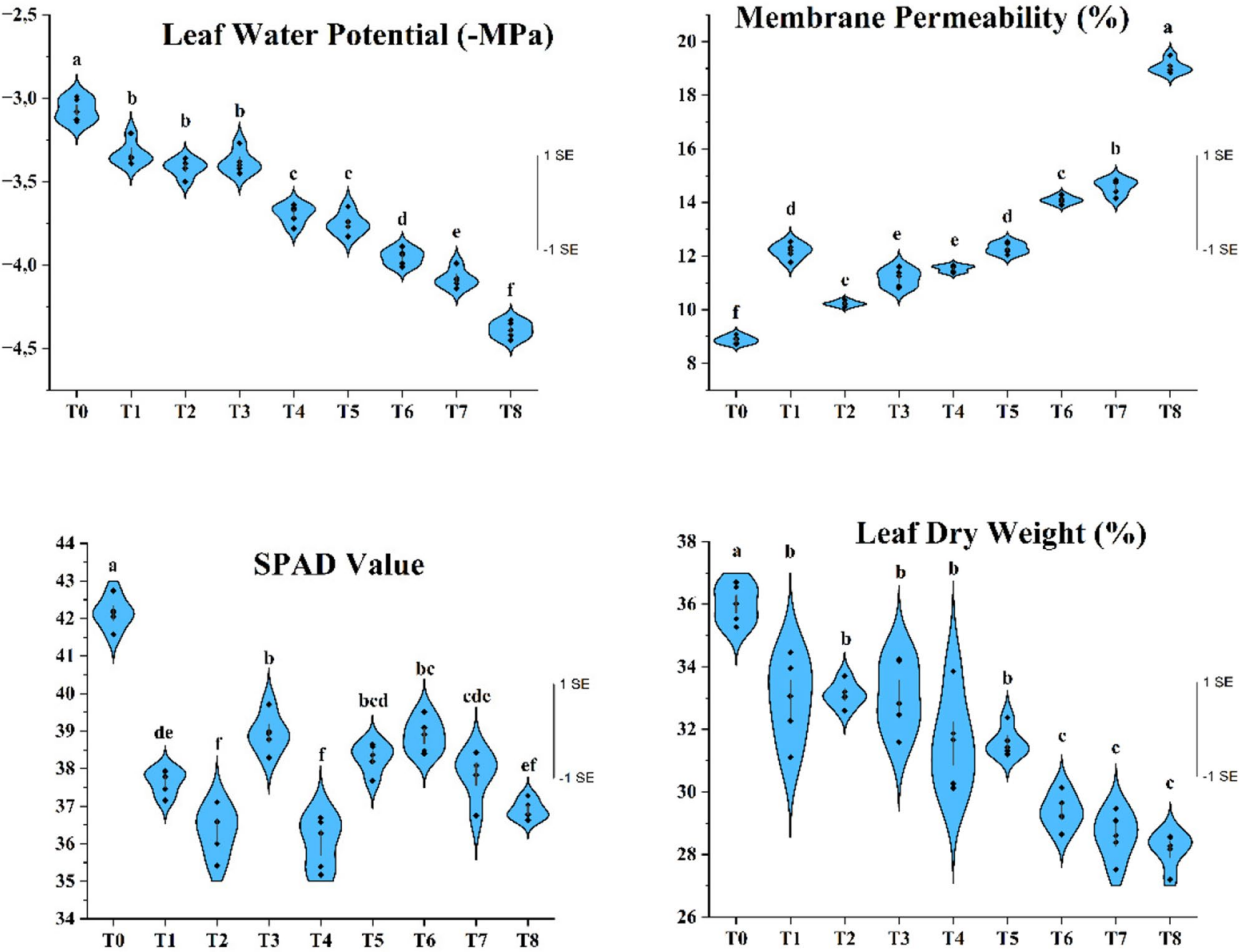
Effects of the irrigation treatments on physiological properties. Values are presented as mean ± SE (n = 5), with samples taken from the pooled canopy of all trees within each treatment (9 trees in total), and data pooled across 2021–2023. Different letters indicate statistically significant differences among irrigation treatments according to Tukey’s multiple comparison test (P ≤ 0.05)

SPAD values, reflecting leaf chlorophyll content, also differed among treatments (Fig. 7). The highest three-year mean occurred in T_0_ (42.14), while drought-affected treatments consistently showed lower SPAD values. Irrigation applied after drought did not always restore chlorophyll content to control levels, suggesting delayed physiological recovery and a cumulative effect of prior-year stress. Supplemental irrigation during autumn and winter partially reduced SPAD loss, indicating some off-season plasticity. Declines in chlorophyll under drought are widely associated with enhanced chlorophyllase activity [73], inhibited biosynthesis [74], and ROS-mediated chloroplast injury leading to photooxidation [75, 76, 77]. Restricted nitrogen uptake under water deficit may further limit chlorophyll production [78]. Although chlorophyll degradation is typically viewed as stress damage, some studies note its potential photoprotective role by reducing light absorption under excess excitation energy [79]. The consistently lower SPAD values in drought treatments in our study align with extensive reports across almond, mandarin, mango, pomegranate, plum, apple, peach, apricot, grapevine, cherry, olive and orange [34, 37, 45, 46, 50, 64, 80–87, 88].

Relative leaf dry weight showed a clear response to irrigation treatments, with the highest value in T_0_ (36.01%) and the lowest in T_6_ (29.37%), T_7_ (28.61%) and T_8_ (28.15%). These reductions reflect the combined effects of drought on cell division, leaf expansion and photosynthetic capacity, all of which limit dry matter production under water deficit [89, 90]. Thus, the lower relative leaf dry weight in drought-stressed treatments represents direct constraints on biomass accumulation and aligns with the well-documented inhibitory impact of drought on growth and carbon assimilation.

### Biochemical analysis

Under drought and other environmental stresses, plants inevitably generate reactive oxygen species (ROS), including singlet oxygen(^1^O_2_), superoxide ($\:{\mathrm{O}}_{\mathrm{2}}^{\mathrm{-}}$), hydrogen peroxide (H_2_O_2_) and hydroxyl radicals($\:{{}_{\text{}}{}^{\mathrm{.}}\mathrm{OH}}_{\mathrm{-}}$). Although ROS play essential roles in signaling related to growth, defense and stress responses, their excessive accumulation disrupts redox homeostasis and causes oxidative damage to cellular components [91–93, 94]. Antioxidant enzymes such as SOD, CAT and POD constitute the main detoxification system, with SOD converting superoxide to H_2_O_2_ and CAT and POD subsequently degrading H_2_O_2_ [95, 96].

**Fig. 8:**
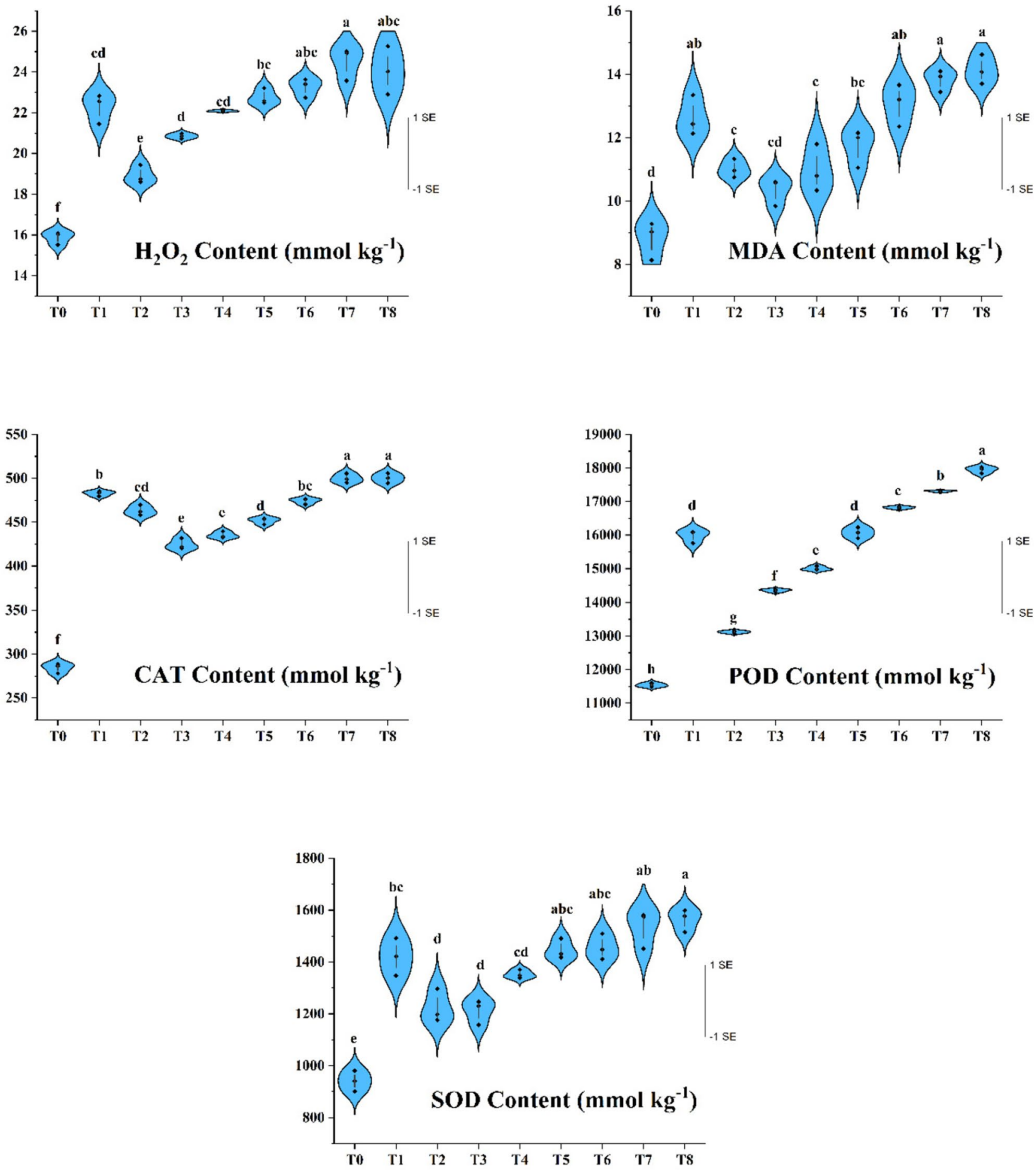
Effects of the irrigation treatments on biochemical properties, values are presented as mean ± SE (n= 3 per treatment), with data pooled across 2021–2023. Different letters indicate statistically significant differences among irrigation treatments according to Tukey’s multiple comparison test (P ≤ 0.05)

Irrigation regimes significantly affected H_2_O_2_ concentrations in apricot leaves (Fig. 8). T_0_, representing full irrigation, showed the lowest H_2_O_2_ content (15.88 mmol kg⁻¹), whereas T_6_, T_7_ and T_8_ each subjected to prolonged or delayed water restriction displayed markedly higher levels (23.25–24.50 mmol kg⁻¹). These increases reflect strong oxidative pressure under severe drought and indicate that both the intensity and timing of water deficit strongly influence ROS accumulation. Elevated H_2_O_2_ under T_6_–T_8_ suggests that metabolic resources normally supporting growth and reproduction were diverted toward protective oxidative defense. Similar H_2_O_2_ increases under drought have been reported in pistachio, apple, grapevine, olive and tangerine [38, 85, 97–99].

Malondialdehyde (MDA), a marker of lipid peroxidation and membrane injury [100], also increased sharply under drought. The highest MDA levels occurred in T_6_–T_8_ and T_1_ (12.63–14.13 mmol kg⁻¹), while the lowest were found in T_0_ (8.81 mmol kg⁻¹) and T_3_ (10.34 mmol kg⁻¹). These patterns indicate intensified membrane degradation under water scarcity. Elevated MDA levels in drought treatments point to substantial oxidative damage to membrane lipids, which compromises compartmentation and ion homeostasis and constrains growth and reproductive processes. These findings align with numerous earlier reports in pistachio, pomegranate, olive, grapevine, peanut, mulberry and apple [34, 38, 70, 71, 81, 85, 97–103].

Antioxidant enzymes showed strong activation under drought. CAT activity was lowest in T_0_ (284.37 nmol kg⁻¹) and highest in T_8_ and T_7_ (500.16 and 499.85 nmol kg⁻¹, respectively). POD followed a similar pattern, with maximum activity in T_8_ (17,942 nmol kg⁻¹) and minimum in T_0_ (11,538 nmol kg⁻¹). SOD activity was likewise lowest in T_0_ (940.76 nmol kg⁻¹) and highest in T_8_, T_7_, T6 and T_5_ (1446–1563 nmol kg⁻¹). These increases indicate enhanced detoxification of superoxide radicals and activation of the antioxidant machinery in response to intensified oxidative stress.

Overall, the higher H_2_O_2_ and MDA levels together with increased SOD, CAT and POD activities demonstrate a strong induction of antioxidative defenses under severe drought (especially T_5_–T_8_). However, the concurrent rise in MDA shows that antioxidant activation is only partially effective, and oxidative damage still accumulates under prolonged water deficit. This suggests that severe drought diverts metabolic resources from growth and reproductive development toward protective functions. These results align with previous studies in Pistacia species [32, 104] and other fruit crops including apple, plum, pomegranate, olive and pistachio [37, 99, 102, 105, 106].

In summary, both the severity and timing of drought strongly shape ROS accumulation and antioxidant enzyme activity in apricot. Although antioxidant responses increase in an attempt to counteract oxidative stress, they do not fully restore redox balance under prolonged deficit. The dependence of these biochemical traits on irrigation strategy highlights the crucial role of appropriate water management in reducing oxidative burden and supporting cellular integrity, growth and reproductive performance.

### Correlation between morphological, phenological, physiological and biochemical properties of apricot under water stress

**Fig. 9:**
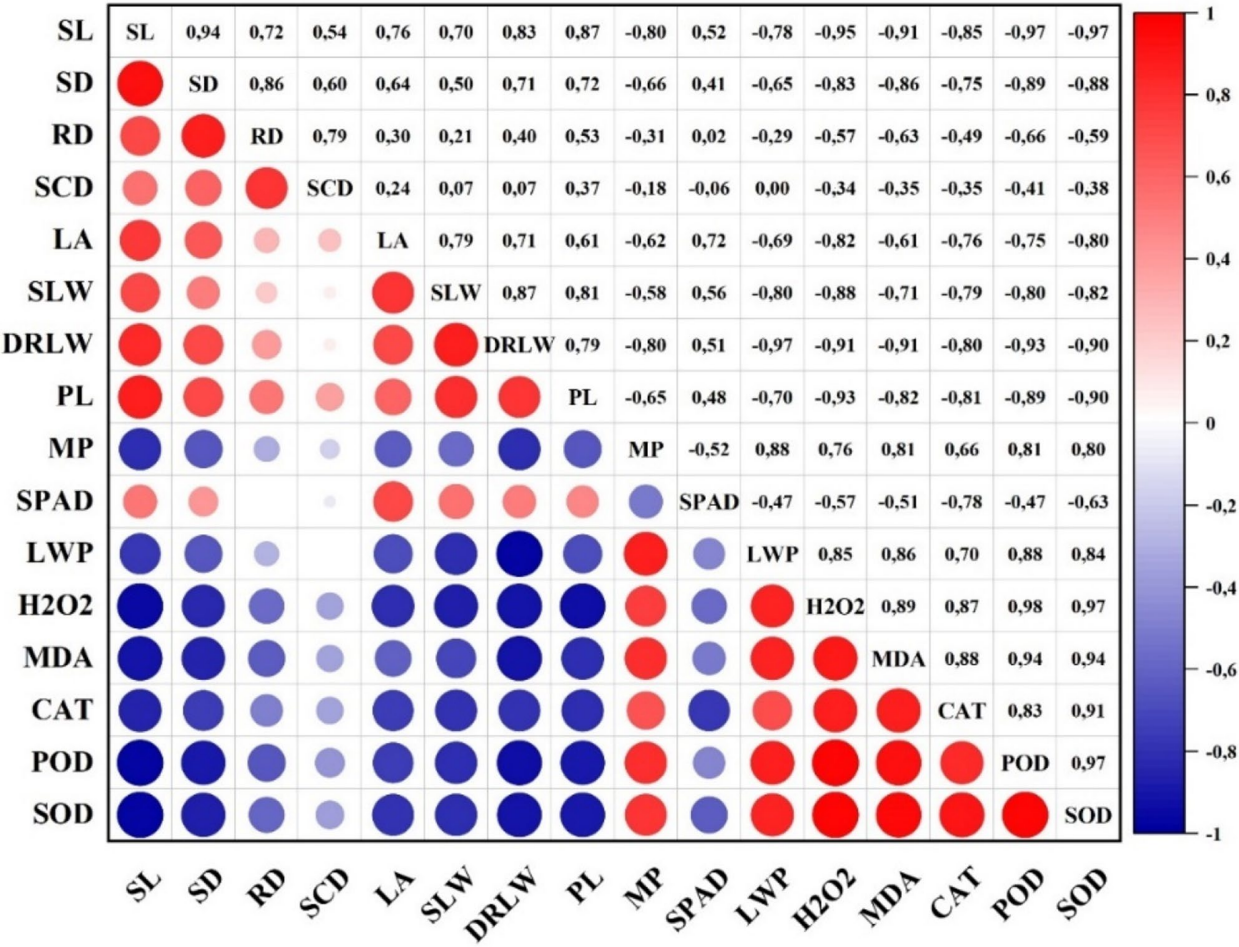
Correlation between features of the young apricot trees under water stress (SL: Shoot Length, SD: Shoot Diameter, RD: Rootstock Diameter, SCD: Scion Diameter, LA: Leaf Area, SLW: Specific Leaf Weight, DRLW: Dried Relative Leaf Weight, PL: Pistil Length, MP: Membrane Permeability, SPAD: SPAD Value, H2O2: Hydrogen Peroxide, MDA: Malondialdehyde, CAT: Catalase, POD: Peroxidase, SOD: Superoxide Dismutase)

Figure 9 summarizes the correlations among morphological, phenological, physiological and biochemical parameters in young apricot trees subjected to different irrigation regimes. Overall, growth-related traits such as shoot length (SL), shoot diameter (SD), root diameter (RD), leaf area (LA), specific leaf weight (SLW) and dry relative leaf weight (DRLW) were strongly and negatively associated with oxidative stress indicators and antioxidant enzyme activities (H_2_O_2_, MDA, SOD, CAT, POD) and membrane permeability (MP), while showing positive relationships with each other. This pattern indicates that increased oxidative stress and membrane damage are closely linked to reductions in vegetative growth under drought conditions.

(SL: Shoot Length, SD: Shoot Diameter, RD: Rootstock Diameter, SCD: Scion Diameter, LA: Leaf Area, SLW: Specific Leaf Weight, DRLW: Dried Relative Leaf Weight, PL: Pistil Length, MP: Membrane Permeability, SPAD: SPAD Value, H2O2: Hydrogen Peroxide, MDA: Malondialdehyde, CAT: Catalase, POD: Peroxidase, SOD: Superoxide Dismutase)

Pistil length (PL) also showed positive correlations with key vegetative traits (e.g. SL, SD, SLW, DRLW), but negative correlations with most stress-related parameters, highlighting that improved water status and lower oxidative load favor both vegetative growth and floral organ development. In contrast, MP, H_2_O_2_, MDA and the activities of SOD, CAT and POD were all positively intercorrelated, forming a coherent stress-response cluster that reflects the joint activation of oxidative and antioxidative processes under water deficit.

Together, these correlations show that irrigation regimes which limit ROS accumulation and membrane damage tend to maintain higher growth and reproductive performance, whereas more severe or poorly timed drought leads to a coordinated increase in oxidative stress markers and a decline in morphological and floral traits. The detailed correlation coefficients for all trait combinations are presented in Fig. 9.

## Conclusion

Our findings demonstrate that irrigation timing is a decisive factor influencing reproductive success in apricot. Water deficits during late summer—particularly in July and August—impose largely irreversible constraints on pistil development, ultimately reducing fruit set and yield. Irrigation applied later in autumn or winter cannot compensate for these developmental disruptions, underscoring the importance of maintaining adequate soil moisture during the post-harvest period when floral initiation and pistil differentiation are active. Prioritizing late-summer irrigation is therefore essential for sustaining productivity in arid and semi-arid orchard systems.

Although this study was conducted under controlled conditions, the findings provide a meaningful framework for understanding how irrigation timing shapes reproductive development in apricot. Additional research across different environments, cultivars, and orchard-scale systems would help extend the applicability of these results to broader production contexts. Exploring the interactions among rootstock characteristics, soil properties, and long-term drought patterns with floral organogenesis will further support the refinement of irrigation strategies for orchards in arid and semi-arid regions.

## Data Availability

The datasets generated and/or analyzed during the current study are available from the corresponding author on reasonable request.
